# Sensing and Impedance Characteristics of YbTaO_4_ Sensing Membranes

**DOI:** 10.1038/s41598-018-30993-7

**Published:** 2018-08-27

**Authors:** Tung-Ming Pan, Yu-Shu Huang, Jim-Long Her

**Affiliations:** 1 Department of Electronics Engineering, Chang Gung University, Taoyuan, 33302 Taiwan; 2 Division of Urology, Chang Gung Memorial Hospital, Taoyuan, 33305 Taiwan; 3 Division of Natural Science, Center for General Education, Chang Gung University, Taoyuan, 33302 Taiwan

## Abstract

In this study we developed ytterbium tantalum oxide (YbTaO_4_) sensing membranes for use in electrolyte–insulator–semiconductor (EIS) pH sensors. The influence of rapid thermal annealing (RTA) treatment on the sensing and impedance properties of the YbTaO_4_ sensing membranes deposited through reactive co-sputtering onto Si substrates was explored. X-ray diffraction, atomic force microscopy, and X-ray photoelectron spectroscopy revealed the structural, morphological, and chemical features, respectively, of these YbTaO_4_ films annealed at 700, 800 and 900 °C. The YbTaO_4_ EIS device annealed at the 800 °C exhibited a super-Nernstian response of 71.17 mV/pH within the pH range of 2–12. It also showed the lowest hysteresis voltage ( < 1 mV) and the lowest drift rate (0.22 mV/h) among the tested systems. Presumably, the optimal annealing temperature improved the stoichiometry of YbTaO_4_ film and increased its (−131)-oriented nanograin size. Moreover, the impedance properties of YbTaO_4_ EIS sensors were investigated by using the capacitance–voltage method. The resistance and capacitance of YbTaO_4_ sensing films annealed at three various temperatures were evaluated by using different frequency ranges in accumulation, depletion, and inversion regions. The semicircle diameter of the YbTaO_4_ EIS sensor became smaller, due to a gradual decrease in the bulk resistance of the EIS device, as the RTA temperature was increased.

## Introduction

An ion-sensitive field-effect-transistor (ISFET) device was first developed by Bergveld in 1970 as a replacement for a fragile glass electrode^1^. Over the past four decades, research on various kinds of ISFET-based biosensors, for example glucose, urea, protein, DNA hybridization, and DNA methylation detection^2–7^, has been conducted because they have the advantages of fast response, small size, and low cost. In general, the gate electrode of a conventional metal-oxide-semiconductor field-effect transistor (MOSFET) is replaced by a chemically sensitive oxide layer, an electrolyte, and a reference electrode to become an ISFET device. An electrolyte-insulator-semiconductor (EIS) capacitor has a simple structure and easy fabrication with respect to an ISFET device. The surface potential of such an ISFET is modulated by the change in the charges at the interface between the oxide layer and electrolyte, thus leading to the shift of the threshold voltage and the variation of the drain-source current. As a result, quality and chemical stability of the sensing film play a key role in the applications of an ISFET or EIS device. The most widely used sensitive oxide film is SiO_2_ in ISFETs, but its application is severely limited by its poor sensitivity^8^. Therefore, Si_3_N_4_, Al_2_O_3_, Ta_2_O_5_, TiO_2_, ZrO_2_, and HfO_2_ films were investigated as alternative sensitive oxides in ISFETs or EISs to improve their sensitivities^9–12^. However, the pH sensitivity of the traditional ISFET or EIS sensors could not exceed the Nernstian limit (59.18 mV/pH at 25 °C) because of intrinsic properties of these metal oxide films. In addition, there are some materials related problems with silicide at the interface of oxide film/Si substrate after thermal treatment and dangling bonds on the sensing film during the thin-film deposition to impact their sensing performance^13,14^.

In search of the low defect density and high thermal stability in high dielectric constant (κ) metal oxide films, rare-earth (RE) oxide thin films have been studied for use as a replacement MOSFET gate dielectric due to their high κ values, large bandgap energies, good thermodynamic properties, high resistivities, and high conduction-band offsets^15,16^. Of these RE oxides, ytterbium oxide (Yb_2_O_3_) thin film turns out to be a potential gate oxide because of its novel properties, including excellent thermal and chemical stability, large bandgap (~5 eV), and high κ (~15), in which the κ values depend on deposition processing^17,18^. Nevertheless, due to the highly hygroscopic nature, there is a need to develop the fabrication of stable RE oxide film, suppressing the formation of a hydroxide layer on the film because of the presence of oxygen vacancies^19^. The formation of oxygen vacancies can contribute to the occurrence of a sufficient atomic reorganization in the film structure. In order to solve the aforementioned shortcoming, in an effort to remove the oxygen vacancies in the RE oxide film, the addition of Ti or TiO_2_ into the film could be reduced the moisture absorption of RE oxides, thereby improving the structural and electrical properties^20,21^. Recently, our group previously demonstrated the structural and sensing characteristics of Yb_2_Ti_2_O_7_ sensing films in an EIS sensor^22^, in contrast, the resistance to the level of moisture absorption for the incorporation of Ta into the Yb_2_O_3_ film after post-annealing treatment to eliminate the oxygen vacancies is still unclear. The aim of this paper is to explore the effect of post-annealing treatment on the structural, sensing, and impedance characteristics of ytterbium tantalum oxide (YbTaO_4_) sensing films deposited on Si substrates through reactive rf co-sputtering. X-ray diffraction (XRD), atomic force microscope (AFM), and X-ray photoelectron spectroscopy (XPS) were employed to examine the film structures, surface morphologies, and chemical compositions of YbTaO_4_ films annealed at three different temperatures (700, 800 and 900 °C), respectively. In addition, structural characteristics of the YbTaO_4_ films are correlated to sensing and impedance properties of the EIS sensors after annealing at three temperatures. In this study, the YbTiO_4_ membrane after RTA at 800 °C showed a higher pH sensitivity (71.17 mV/pH), a smaller hysteresis voltage (<1 mV) and a lower drift rate (0.22 mV/h), compared with other RTA temperatures.

## Methods

### Fabrication

Prior to the deposition of sensing film, the Si substrate was cleaned and hydrogen-terminated by using diluted HF. The YbTa_x_O_y_ thin films were grown on p-Si (100) substrates with a resistivity of 5–10 Ω-cm by rf co-sputtering using from metal Yb and Ta as target materials in a mixture of Ar/O_2_ (5 sccm/20 sccm). The plasma power of Yb and Ta targets was 100 W. The chamber pressure was 1 × 10^−3^ Torr during the growth process and the substrate temperature was 27 °C. The growth rate and physical thickness of the YbTa_x_O_y_ film were ~2 nm/min and ~60 nm, respectively. Subsequently, the samples were annealed at three various temperatures (700, 800 and 900 °C) by rapid thermal annealing (RTA) in oxygen (O_2_) ambient for 30 s to form an YbTaO_4_ compound. Next, the back-side oxide of Si wafer was etched by buffer oxide etchant (BOE) solution and Al (400 nm thick) as a back-side electrode was deposited by thermal evaporation to achieve good electrical contact. The sensing area of 3.14 mm^2^ was defined by a robotic dispensing system using an adhesive silicone gel (S181) as an isolating layer. The EIS device was mounted on the Cu-coated printed circuit board (PCB) with an Al back-side contact through Ag paste. Finally, the non-sensing region of the EIS sensor was covered with epoxy.

### Characterization

The orientation and phase of these films were investigated by XRD (Bruker D8 discover diffractometer) in a scan range of 2θ = 20°–60° using a step time of 1 s and a step size of 0.05°. The Cu K_α_ radiation (λ = 1.5406 Å) was run under a voltage of 40 kV and a current of 20 mA. A tapping mode AFM (NT-MDT Solver P47) was employed to explore the surface topography and determine the surface roughness (root-mean-square, R_rms_) of the YbTaO_4_ films annealed at three temperatures. The R_rms_ roughness of these samples was estimated in scanning areas of 3 × 3 μm^2^. The film composition of YbTaO_4_ films was analyzed using XPS (Thermo VG Scientific Microlab 350 system) with a monochromatic Al K_α_ source (1486.7 eV). The binding energy scale of each element was calibrated by setting the main hydrocarbon peak at a binding energy of 285 eV (C 1 s).

### Measurement

The pH sensitivity, hysteresis voltage, and drift rate of the YbTaO_4_ EIS devices were evaluated by capacitance–voltage (C–V) curves. The C–V measurements for different pH buffer solutions (Merck Inc.) were performed using inductance–capacitance–resistance (LCR) meter (Agilent 4284 A), operated at 500 Hz with an applied ac voltage of 10 mV. The Ag/AgCl electrode (commercial liquid-junction electrode) electrode as a reference electrode was used. The impedance measurement of EIS devices was performed by a combination of Agilent 4284 A and 4285 A LCR meters. To achieve the steady results, all samples were kept in reverse osmosis (RO) water for 24 h before measurement. The sensing membrane was washed with deionized water before transferring to subsequent pH solution. In order to prevent light and noise interference, all the measurements were performed in a dark box. Three devices on each sample were repeated at least three times to measure sensing performance of the YbTaO_4_ EIS sensors.

## Results and Discussion

### Structural properties of YbTaO_4_ sensing films

**Figure 1:**
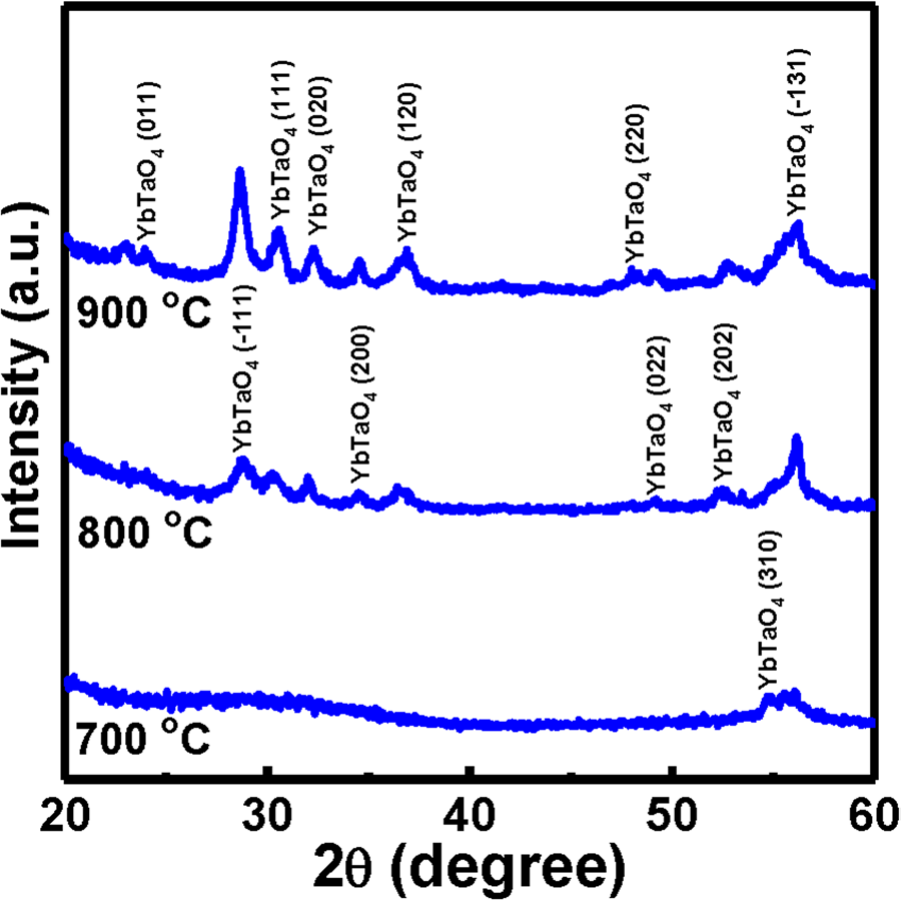
XRD patterns of YbTaO_4_ films annealed at three different RTA temperatures.

The XRD patterns of all YbTaO_4_ films deposited on a Si substrate after RTA at different temperatures (700–900 °C) in O_2_ ambient were presented in Fig. 1. The YbTaO_4_ films after RTA at three temperatures have a monoclinic structure. The YbTaO_4_ diffraction peaks were recorded at (310) and (−131) peaks for the film annealed at 700 °C. Apart from the increment in the intensity of the existing YbTaO_4_ peaks, an additional YbTaO_4_ (011), (−111), (111), (020), (200), (120), (022), and (202) peaks (JCPDS: 00-024-1416) were detected when the annealing temperature was raised up to 800 °C, suggesting a polycrystalline structure. These peaks became more noticeable as the annealing temperature increased. By further increasing the annealing temperature to 900 °C, the increment in the intensity of YbTaO_4_ (−111), (111), (020), (200), and (120) peaks was observed. Overall, as the RTA temperature increased from 700 °C to 800 °C, the intensity of the YbTaO_4_ (−131) peak increased. This condition may indicate the reduction of oxygen vacancies present in the film due to the highest stability offered by (−131)-oriented YbTaO_4_. The crystallite size (or grain size) of the YbTaO_4_ film after three RTA temperatures is determined by using the Scherrer’s equation for the main diffraction peak^23^. The average grain size was calculated to be 0.12, 0.21, and 0.24 nm for the sample annealed at 700, 800 and 900 °C, respectively. Moreover, the crystallite size of the (−131) peak for the YbTaO_4_ film annealed at 700, 800 and 900 °C was 0.12, 0.35 and 0. 19 nm, respectively.

**Figure 2:**
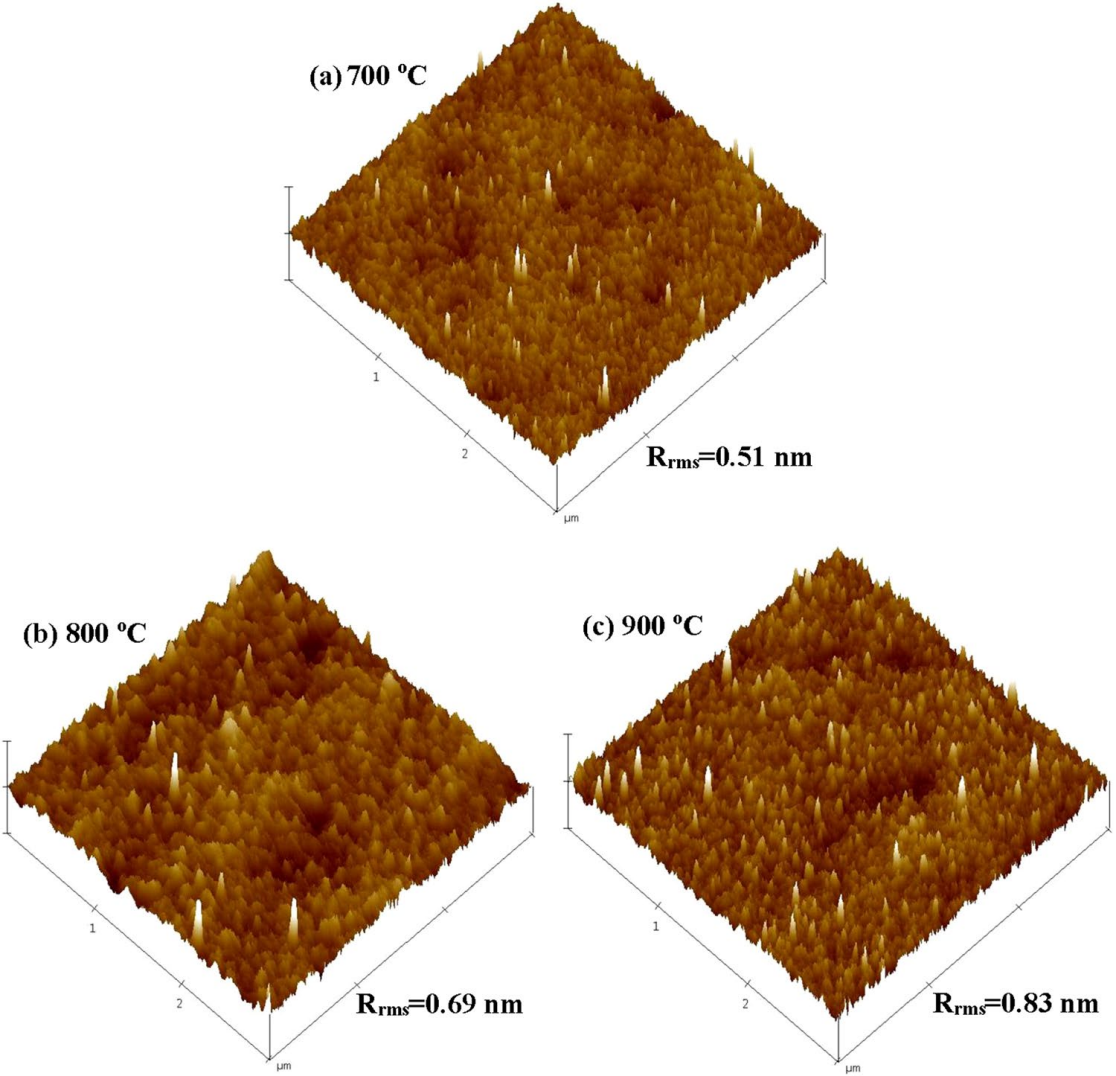
AFM images of YbTaO_4_ films annealed at three RTA temperatures: (**a**) 700 °C, (**b**) 800 °C and (**c**) 900 °C.

The surface morphology of YbTaO_4_ thin films deposited on a Si substrate was evaluated by AFM to examine the effect of RTA temperatures. Figure 2 depicts AFM 3-D surface topographies and R_rms_ roughnesses of the YbTaO_4_ films annealed at various temperatures. In general, the sample is uniform and smooth without defects, such as a crack or void. It can be observed in Fig. 2 that bubblelike grains were developed for all samples. When the film was annealed at 900 °C, more bubblelike grains were formed in contrast to the film annealed at 800 and 700 °C. The R_rms_ roughness of the YbTaO_4_ films annealed at 700, 800 and 900 °C was 0.51, 0.69 and 0.83 nm, respectively. The R_rms_ roughness of the sample is in the increasing trend as the RTA temperature increases. The reason of increase of surface roughness might be attributed to the growth of grain size due to the formation of grain agglomerations in the film after annealing at high temperature in oxygen gas. This result indicates that the grain size increases with increasing RTA temperature, as expected, leading to the enhanced crystallinity and the sharpened diffraction peaks. Therefore, the obtained R_rms_ roughness from AFM matches with the XRD results. Hence, more YbTaO_4_ was formed, and a noticeable change in the surface morphology was observed for the sample annealed at 900 °C.

**Figure 3:**
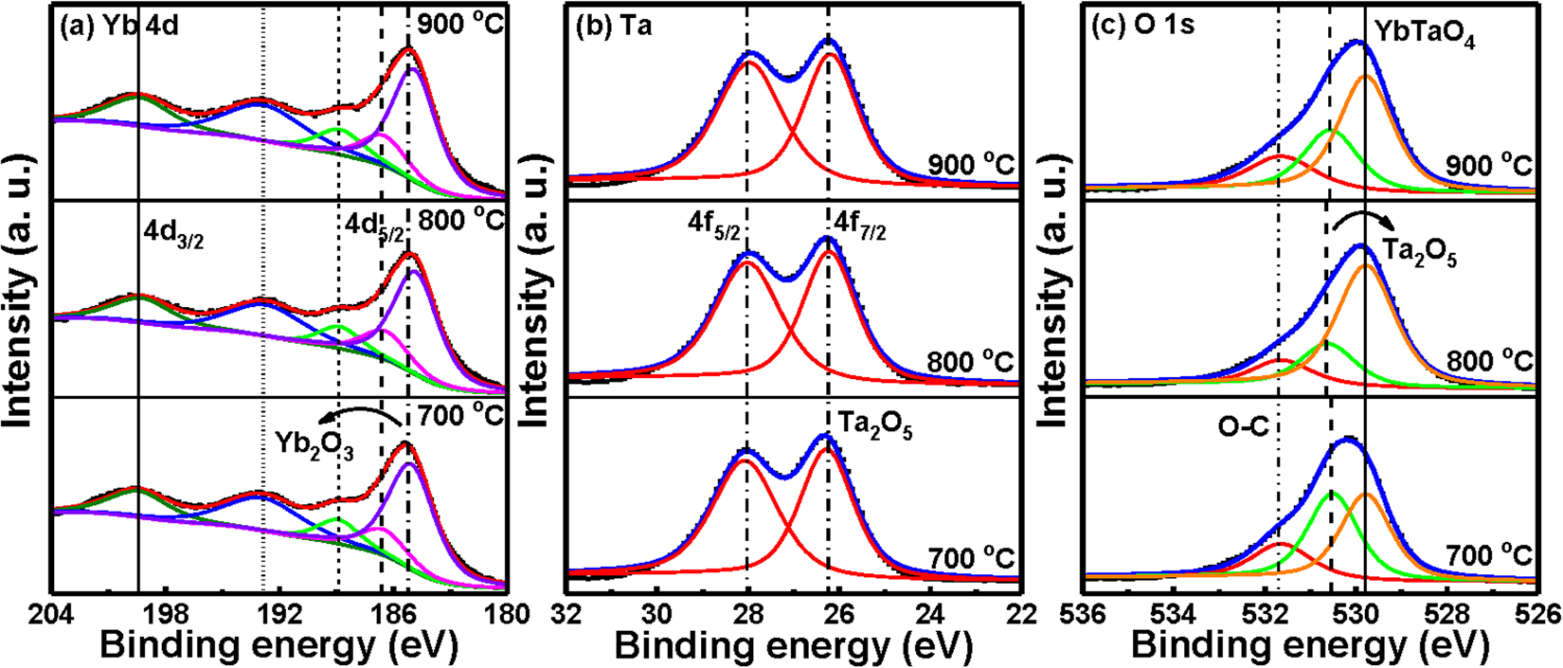
XPS spectra displaying the (**a**) Yb 3d, (**b**) Ta 4 f, and (**c**) O 1 s energy levels in YbTaO_4_ films annealed at three RTA temperatures.

The valence states of Yb, Ta, and O elements in a YbTaO_4_ layer were analyzed by XPS with Ar^+^ ion etching. The 30 s etching process was conducted to remove the surface contamination. The obtained Yb 3d, Ta 4 f and O 1 s spectra of the YbTaO_4_ films after RTA at three temperatures were shown in Fig. 3. After the subtraction of Shirley-type backgrounds, the raw data were all fitted by Gaussian-Lorentzian line shapes. To determine the valence state of Yb in the films annealed at various temperatures, we investigated the XPS spectra of the Yb 4d level in the YbTaO_4_. The ytterbium compounds have two valence states. The filled 4 f shell of divalent ytterbium (Yb^2+^) in the 4d spectrum is a doublet with a peak area ratio of 3:2, while partially filled 4 f shell of trivalent ytterbium (Yb^3+^) is a multiplet^24^. The electronic structure of the Yb 4d multiplet peaks in these films is in good agreement with multiplet splitting in the Yb 4d spectrum of Yb_2_O_3_, which is different from the 4d doublet of Yb^2+^. There are two main Yb 4d_3/2_ and 4d_5/2_ peaks centered at 199.5 ± 0.1 eV and 185.3 ± 0.1 eV, respectively, which are consistent with the binding energy of Yb 4d for Yb_2_O_3_ ref.^25^. The Yb 4d_5/2_ peak of the film annealed at 800 and 900 °C was slightly shifted toward a higher binding energy, suggesting the incorporation of more Ta atom into the Yb_2_O_3_ film forming a YbTaO_4_ compound. Figure 3(b) shows the double peak features in the Ta 4f XPS spectra for YbTaO_4_ films annealed at three temperatures. The Ta 4f_5/2_ and 4f_7/2_ peaks located at binding energies of 28.1 and 26.2 eV, respectively, are assigned to pure Ta_2_O_5_ film^26^. The spin-orbit splitting energy of Ta_2_O_5_ film was 1.9 eV from Ta 4f_5/2_ to Ta 4f_7/2_ peak. The deconvolution results of these YbTaO_4_ films demonstrated a perfect fit for Ta 4f_5/2_ and 4f_7/2_ peaks located at two 28 ± 0.1 and 26.1 ± 0.1 eV and splitting energy of ~1.9 eV, which are in good agreement with the reported values for Ta_2_O_5_ ref.^26^ indicating that the Ta exhibits the highest oxidation state. These sensing films consist of tantalum suboxides (Ta_2_O_5_) with no TaO_x_ content. The splitting energy of about 1.9 eV can consider strong binding interaction between the tantalum and the oxygen atoms. The reaction of Ta_2_O_5_ with Yb_2_O_3_ can form a YbTa_x_O_y_ compound. The O 1 s XPS spectra of the YbTaO_4_ films after RTA at different temperatures were deconvoluted with Gaussian-Lorentzian curve fitting after the Shirley background, as shown in Fig. 3(c). The fitting results depicted three peaks located at 531.6, ~530.5, and 529.8 eV. The high binding-energy peak of 531.6 eV corresponds to the surface contamination is caused by adsorption of atmospheric carbon-containing species. The median binding-energy peak of 530.5 eV can be related to the O^2−^ species occurring in Ta_2_O_5_^26^. The low binding-energy peak of 529.8 eV can be attributed to the Yb-O-Ta bonding. These low peak intensities are assigned to O-C (or O=C) bonds resulting from various species present in the organic carbon overlayer on top of the YbTaO_4_ films. The peak intensities of YbTaO_4_ and Ta_2_O_5_ components for the film annealed at 800 °C exhibited higher and lower, respectively, in the O 1 s signal compared with other temperatures. In addition, the O 1 s peak corresponding to YbTaO_4_ for the sample annealed at 900 °C was a lower intensity than that at 800 °C. During high-temperature annealing, the more Ta or/and Yb atoms diffuse readily from the YbTaO_4_ film to form a thicker silicate layer at the YbTaO_4_–Si interface^14,15^.

### Sensing and impedance characteristics of YbTaO_4_ EIS sensors

After the film material analyses, the sensing performance (pH sensitivity, hysteresis voltage and drift rate) of YbTaO_4_ EIS sensors after RTA at three temperatures was investigated. The flatband voltage (*V*_*FB*_) of an ISFET or EIS device can be expressed as:^27^

1 $${{V}}_{{FB}}={{E}}_{{ref}}-{{\psi }}_{{\rm{0}}}+{{\chi }}^{{sol}}-\frac{{{\rm{\Phi }}}_{{Semi}}}{{q}}-\frac{{{Q}}_{{eff}}}{{{C}}_{{ox}}}$$

where *E*_*ref*_ is the reference electrode potential, *ψ*_0_ is the surface potential, *χ*^*sol*^ is the surface dipole potential of the solution, *Φ*_*Semi*_ is the semiconductor work function, *q* is the elementary charge, *Q*_*eff*_ is the effective charge produced at the oxide-semiconductor interface and in the oxide by the different types of charges (e.g., interface trapped charge, fixed oxide charge, oxide trapped charge), and *C*_*ox*_ is the gate oxide capacitance. Apart from *ψ*_0_, all these terms are fixed values. This term causes an ISFET or EIS device responsive to the tested solution as a result of the polarization and formation of the potential barrier, which is related to the H^+^ concentration. Therefore, an ISFET or EIS device can be sensitive to the solution pH. The potential of electrolyte-insulator interface corresponding to pH can be explained by using a combination of the site-binding model and the Gouy–Chapman–Stern theory^28^. The pH sensitivity of an ISFET device is derived from Bergveld^28^ as follows:

2 $$\frac{\delta {\psi }_{0}}{\delta {p}{{H}}_{{s}}}=2.303\alpha \frac{{{k}}_{{B}}{T}}{{q}},\,{with}\,{\alpha }=\frac{1}{1+\frac{2.303{{k}}_{{B}}{T}{{C}}_{{dif}}}{{{q}}^{2}{{\beta }}_{{int}}}}$$

where *α* represents a dimensionless sensitivity parameter which varies between 0 and 1, *C*_*diff*_ indicates the differential capacitance of the electric double-layer, and *β*_*int*_ stands for the intrinsic buffer capacity of the oxide surface. The above equation gives information that the *β*_*int*_ and *C*_*diff*_ are effectively influenced the pH sensitivity of an ISFET device. The *C*_*diff*_ is obtained from the Gouy-Chapman-Stern model. In addition, the *β*_*int*_ is related to the density of binding sites on the gate oxide. The higher the intrinsic buffer capacity *β*_*int*_ is, the higher the pH sensitivity of the sensing film will achieve. The intrinsic buffer capacity is associated with the surface roughness and the material quality of the film. The change in the pH value of the electrolyte solution could give rise to a shift of the flatband voltage in the C-V curves.

**Figure 4:**
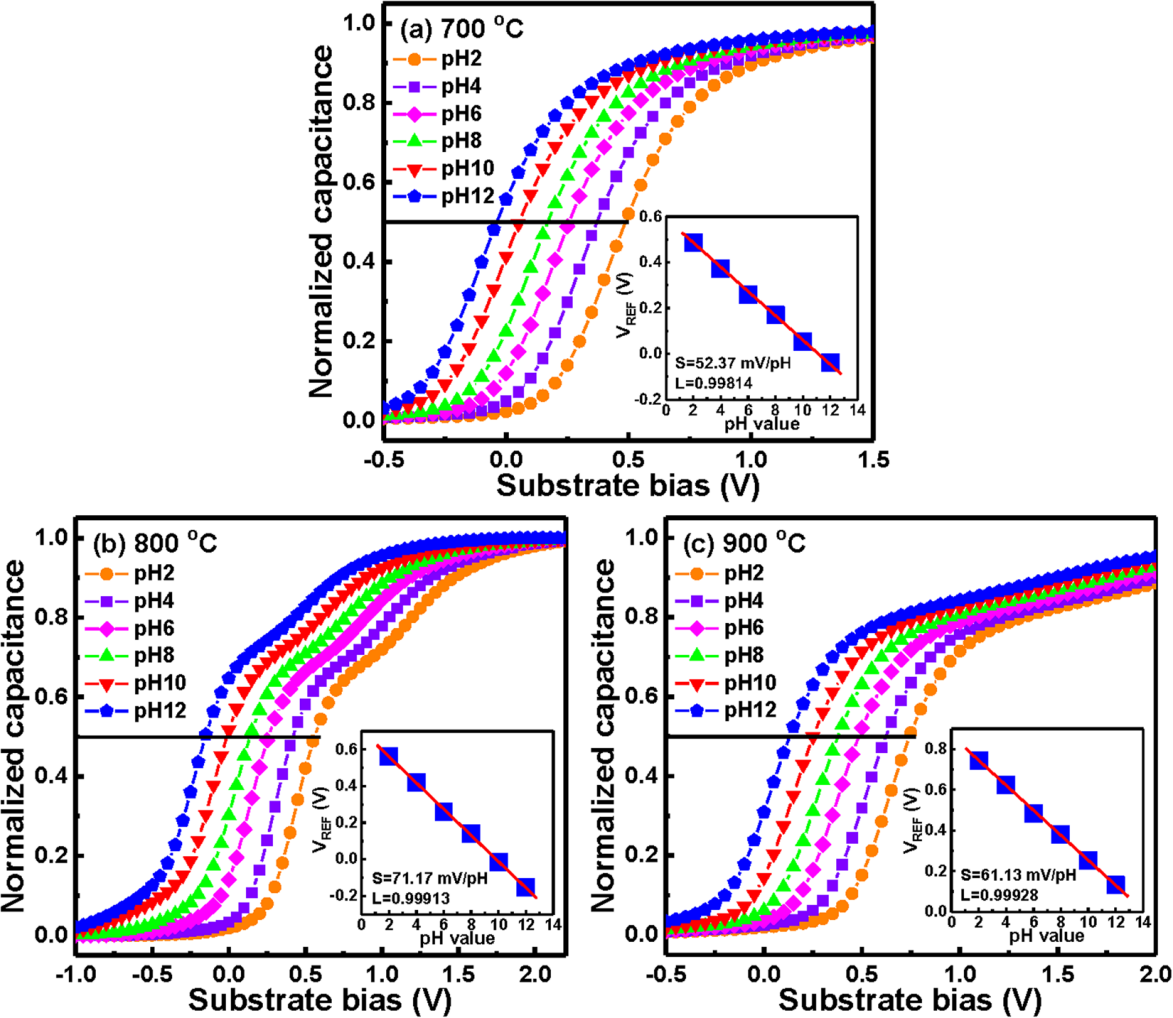
Responses of the *C*–*V* curves after inserting the YbTaO_4_ sensors annealed at three RTA temperatures of (**a**) 700 °C, (**b**) 800 °C and (**c**) 900 °C, when submerged into solutions at values of pH ranging from 2 to 12. The insert in figure shows reference voltage of YbTaO_4_ EIS sensors annealed at three RTA temperatures plotted as a function of pH at room temperature.

In order to evaluate pH sensitivity of the YbTaO_4_ EIS sensors after RTA at different temperatures, a set of C-V curves measured in a wide range of pH 2–12 was tested. Figure 4(a–c) show the normalized C-V curves of the YbTaO_4_ EIS devices annealed at 700, 800 and 900 °C, respectively. For a p-type Si substrate, three zones were visible, namely the accumulation, inversion and depletion regions. The accumulation region is caused by a hole channel on the Si surface when a high positive voltage is applied to substrate electrode. In contrast, when a high negative voltage is applied to substrate electrode, an inversion layer of electrons is formed in the inversion region. The C-V measurements around the depletion region were conducted for electrolyte solutions with pH ranging from 2 to 12. It is evidence that the kinks of the EIS sensors annealed at 800 and 900 °C were found in the depletion region of the C-V curves, possibly indicating the presence of interface state at the oxide-substrate^29^. In an ISFET operation, an adequate number of surface hydroxyl (OH) groups is essential on the gate oxide material for pH measurement at the Nernstian limit. The OH groups can protonate (positively charged, OH_2_^+^) or deprotonate (negatively charged, O^−^)^27^, which relies on the solution pH value. Therefore, the surface potential of the gate oxide changes, which can be measured via a capacitance variation of an EIS device. A negative shift of the reference voltage (V_REF_) with increasing pH values of the investigated electrolyte solution reflects a more negatively charged gate oxide surface. The inset of Fig. 4(a–c) depicts the sensitivity and linearity of the YbTaO_4_ EIS devices after RTA at 700, 800 and 900 °C, respectively. The 0.5C_max_ was set as the reference to extract the V_REF_ versus the change of the pH value. The pH sensitivity and linearity of the C-V curves were achieved by linear regression. The YbTaO_4_ EIS device annealed at 800 °C exhibited the highest sensitivity of 71.17 ± 2.36 mV/pH among these RTA temperatures (52.37 ± 3.25 mV/pH for 700 °C and 61.13 ± 2.26 mV/pH for 900 °C). This result is mainly attributed to the film with a stoichiometric YbTaO_4_ structure and a larger grain size of (−131) plane enhancing the pH sensitivity. Furthermore, the optimal RTA temperature in the oxygen ambient might improve the surface and interfacial material quality and increase the intrinsic buffer capacity (*β*_*int*_). Furthermore, the pH sensitivity of the YbTaO_4_ EIS sensor is higher than the theoretical Nernstian value of 59.4 mV/pH at 27 °C. This super-Nernstian value of our EIS sensor could be related to the mechanism of one transferred electron per 1.5 H^+^ ion^30^. The decrease in pH sensitivity after RTA at 900 °C is the decrease of the surface state density of the OH groups^31^.

**Figure 5:**
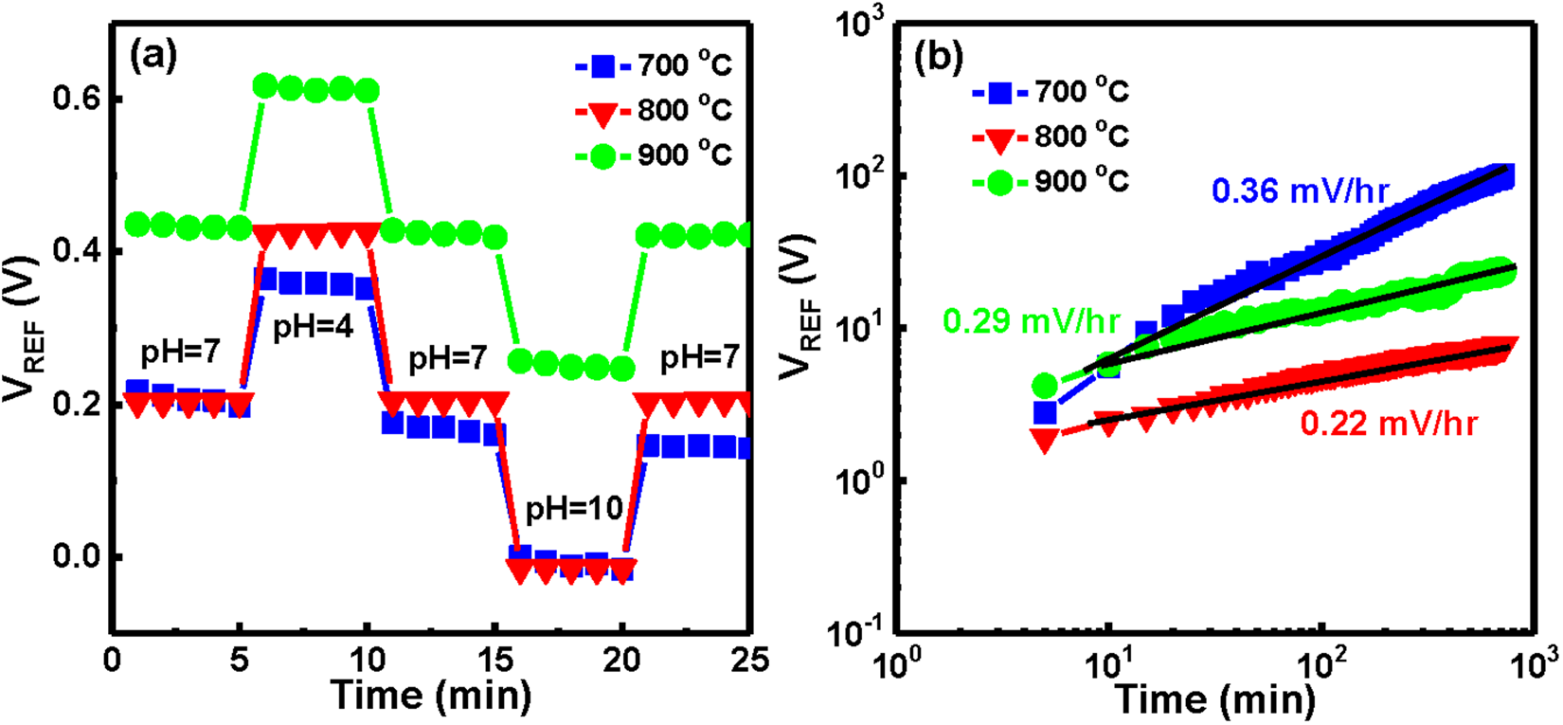
(**a**) Hysteresis curves of YbTaO_4_ EIS sensors annealed at different RTA temperatures during the pH loop of 7→ 4→ 7→ 10→ 7. (**b**) Drift phenomena of YbTaO_4_ EIS devices annealed at three RTA temperatures in a pH = 7 solution.

Furthermore, the hysteresis curves of the YbTaO_4_ EIS sensors with three RTA temperatures were shown in Fig. 5(a). The EIS sensors were submerged in the solutions of the pH loop of 7 → 4 → 7 → 10 → 7. The hysteresis phenomenon might be due to the defects (e.g., oxygen vacancies, dangling bonds) of the film, these defects could react with the hydroxyl groups, thereby leading to hysteresis effect^32^. The YbTaO_4_ EIS device after RTA at 800 °C had the lowest hysteresis voltage of 1 ± 0.2 mV among these annealing temperatures (63 ± 8.9 mV for 700 °C and 11 ± 2.8 mV for 900 °C), suggesting that the optimal RTA temperature could effectively reduce the oxygen vacancies and dangling bonds. On the contrary, the EIS sensor annealed at 700 °C showed a relatively larger hysteresis voltage of 63 mV compared to other temperatures. This result could be attributed to a high number of defects in the YbTaO_4_ film because these defects cannot be removed by the low annealing temperature. Figure 5(b) depicts the drift characteristics of the YbTaO_4_ sensing films annealed at three RTA temperatures. Each of the EIS sensors was measured in a pH 7 buffer solution for a period of time. The change in the V_REF_ can be expressed as ΔV_REF_ = V_REF_(t)–V_REF_(0). The slope of the drift characteristics indicates the drift rate of an EIS device. The drift effect can be interpreted by the hopping and/or trap-limited transport of water-related species^33^, which the localized defects could interact with the tested solution, thus resulting in the gate voltage shift. Figure 5(b) demonstrates that the YbTaO_4_ EIS sensor with the 800 °C had the lowest drift rate of 0.22 ± 0.03 mV/h, whereas the EIS device with the 700 °C featured the highest drift rate of 0.36 ± 0.08 mV/h. The lower drift rate may be due to the fact that the crystal defects could be eliminated by optimal RTA temperature in O_2_ ambient, hence causing a lower capacitance of hydrated layer. In contrast, the higher drift rate could be contributed to the higher capacitance value of hydrated layer. In Table 1, the sensing performance of the YbTiO_4_ membrane is compared with commonly used materials for EIS or ISFET-based sensors such as Ta_2_O_5_^9^, Al_2_O_3_^10^, ZrO_2_^11^, HfO_2_^11^, TiO_2_^34^, SnO_2_^35^, and Yb_2_Ti_2_O_7_^22^. It is found that our YbTiO_4_ membrane demonstrated a higher pH sensitivity (71.17 mV/pH), a smaller hysteresis voltage (<1 mV) and a lower drift rate (0.22 mV/h), relative to those of these materials.

**Table 1:** Comparison of sensing performances (sensitivity, hysteresis voltage, and drift rate) of ISFET and EIS sensors fabricated with Ta_2_O_5_, Al_2_O_3_, ZrO_2_, HfO_2_, TiO_2_, SnO_2_, Yb_2_Ti_2_O_7_, and YbTaO_4_ sensing films.

Sensing membrane	Sensitivity (mV/pH)	Hysteresis (mV)	Drift rate (mV/h)
Ta_2_O_5_^9^	55–58	~ 1	<0.5
Al_2_O_3_^10^	53.23	4	x
ZrO_2_^11^	60	5.26	0.68
HfO_2_^11^	57.5	5.88	0.52
TiO_2_^34^	61.44	x	8.94
SnO_2_^35^	57.36	4.8	6.73
Yb_2_Ti_2_O_7_^22^	61.11	1.6	0.25
This work	71.17	<1	0.22

**Figure 6:**
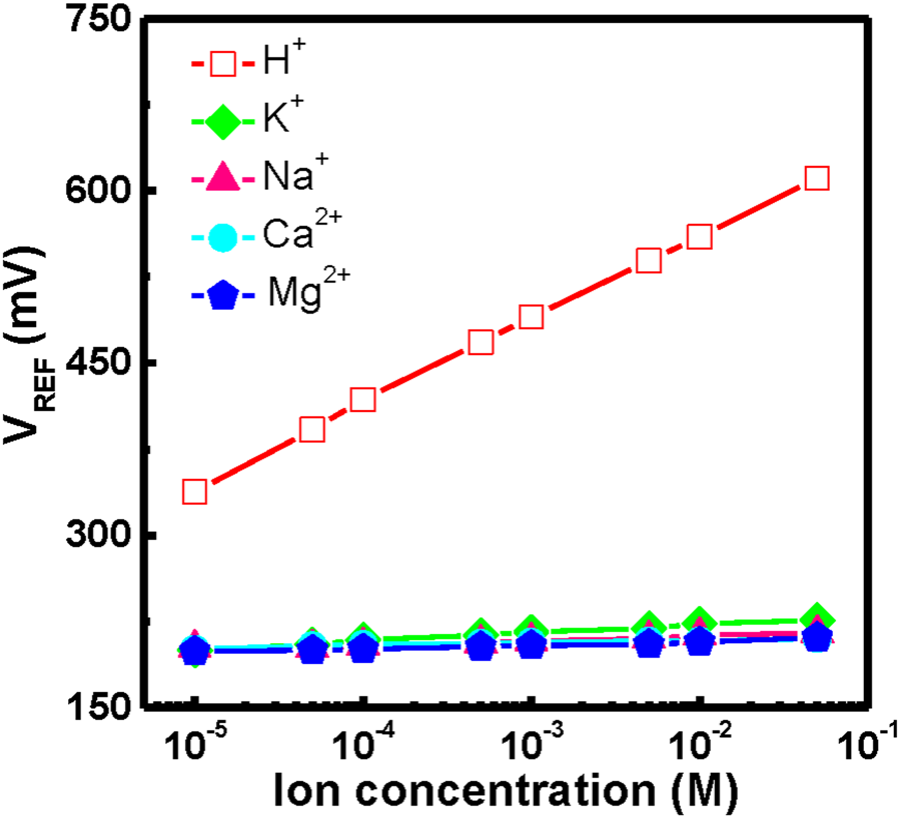
Reference voltage of YbTaO_4_ EIS sensor annealed at 800 °C for different H^+^, K^+^, Na^+^, Ca^2+^, and Mg^2+^ ion concentrations in pH 7.

Figure 6 shows the reference voltage of the YbTiO_4_ EIS sensor annealed at 800 °C for various H^+^, K^+^, Na^+^, Ca^2+^, and Mg^2+^ ion concentrations in pH 7. The response curves were measured in these ion concentrations ranging from 10^−5^ to 0.05 M. It is clear that the YbTiO_4_ sensing membrane had high selectivity to H^+^ ions and less selectivity to other ions, e.g. K^+^, Na^+^, Ca^2+^, Mg^2+^.

**Figure 7:**
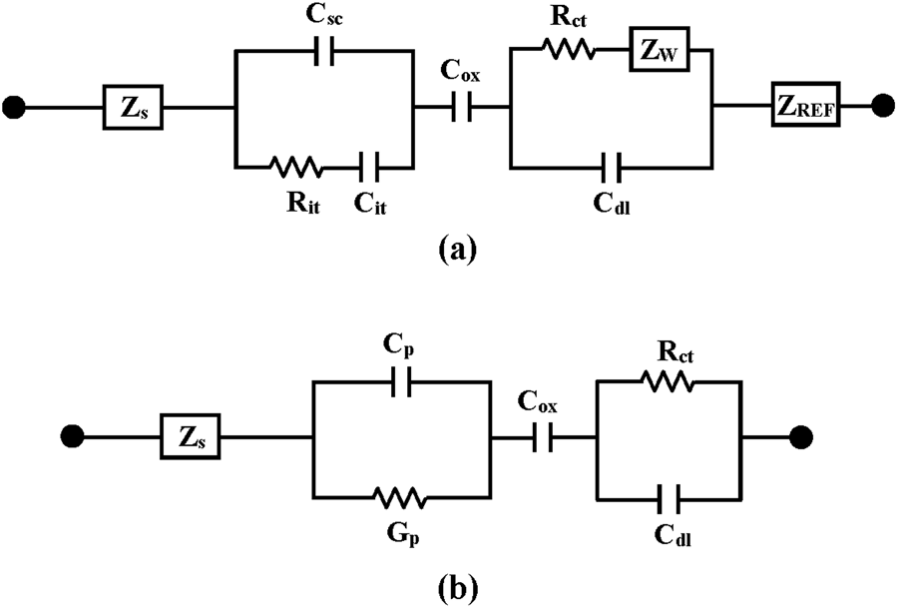
(**a**) Model of circuit elements present when the EIS device is immersed in a pH solution and (**b**) simplified equivalent circuit of an EIS device used in the study. Equivalent elements: Z_s_, Si substrate impedance; C_sc_, space charge capacitance; C_it_, interface trap capacitance; R_it_, interface trap resistance; C_ox_, gate oxide capacitance; C_dl_, double-layer capacitance; R_ct_, charge transfer resistance; Z_W_, Warburg impedance; Z_REF_, reference impedance; C_p_, frequency-dependent capacitance; G_p_, frequency-dependent conductance.

**Figure 8:**
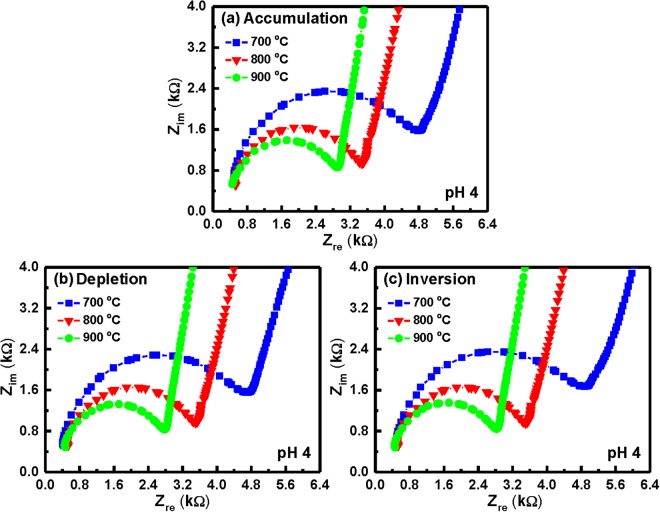
Nyquist diagrams (Z_im_ vs. Z_re_) of the YbTaO_4_ EIS sensors annealed at three RTA temperatures and tested in the (**a**) accumulation, (**b**) depletion and (**c**) inversion regions, when immersed in a pH 4 solution.

**Figure 9:**
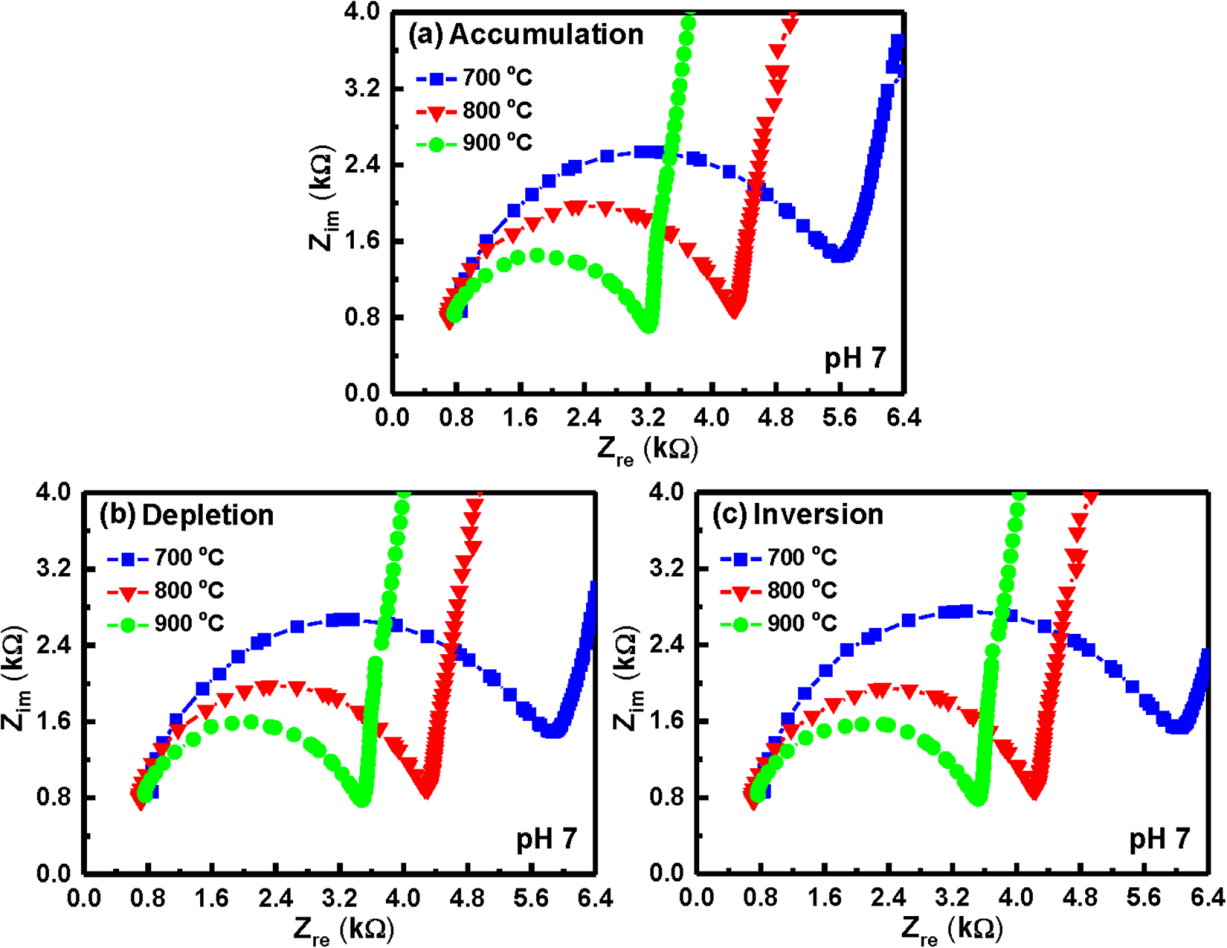
Nyquist diagrams (Z_im_ vs. Z_re_) of the YbTaO_4_ EIS sensors annealed at three RTA temperatures and tested in the (**a**) accumulation, (**b**) depletion and (**c**) inversion regions, when immersed in a pH 7 solution.

**Figure 10:**
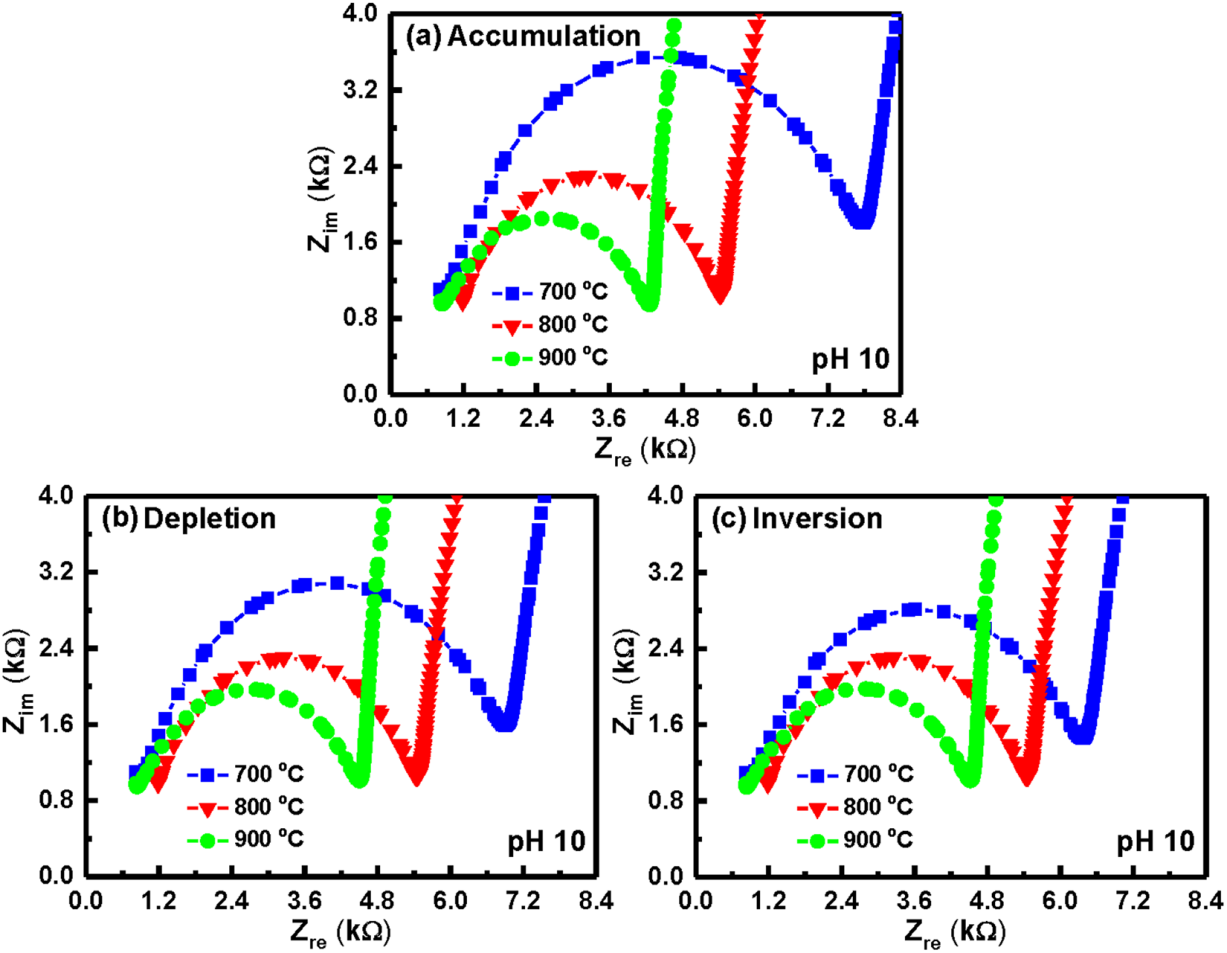
Nyquist diagrams (Z_im_ vs. Z_re_) of the YbTaO_4_ EIS sensors annealed at three RTA temperatures and tested in the (**a**) accumulation, (**b**) depletion and (**c**) inversion regions, when immersed in a pH 10 solution.

Electrochemical impedance spectroscopy is well established as a powerful tool for investigating the mechanisms of electrochemical reactions and measuring the dielectric and transport properties of materials. In addition, it can be successfully applied for the characterization of biosensing surfaces and/or in estimation of bioanalytical signals produced by biosensors. A small sinusoidal perturbation of potential measured is usually applied to an electrochemical device at different frequencies, thus monitoring the variation of electric current or voltage. Figure 7(a) depicts an equivalent circuit of the oxide surface/electrolyte solution interface in an EIS device. The Nyquist plots of the YbTaO_4_ EIS devices annealed at three different RTA temperatures and tested at different solution pH values were presented in Figs 8–10. Each of the plots in all the YbTaO_4_ EIS devices demonstrated a depressed semicircle portion at high frequency region and a slanted straight portion at low frequency region. The results indicate that the semicircle portion is related to charge transfer-limited process and the linear portion is associated with diffusion limited process or mass transfer process at oxide–electrolyte interface. At higher frequency end the intercept corresponds to the Si substrate impedance (Z_S_) and at lower frequency corresponds to the sum of Z_S_ and charge transfer resistance (R_ct_). The value of R_ct_ is a measure of electron transfer across the exposed area of the oxide surface. The conductance method, indicating the loss mechanism because of interface trap capture and emission of carriers, is generally employed to evaluate the density of interface trap states (D_it_) in the depletion region for a MOSFET device^29^. If the capacitance has small losses in the oxide, a simplified equivalent circuit of an EIS sensor for conductance method was shown in Fig. 7(b). The measured conductance originates from the contribution of the interfacial trap states. According to the equivalent circuit of Fig. 7(b), two semicircle features may be observed in Nyquist plots: one is related to the sensing membrane response at higher frequencies, and the other is associated with the oxide–solution interface response at lower frequencies. Figures 8–10 demonstrate that single semicircles were observed in the frequency range 0.2 to 8 MHz for the YbTaO_4_ EIS sensors with different RTA temperatures from 700 to 900 °C.

At pH 4, a semicircle diameter of the YbTaO_4_ EIS sensors annealed at 700 °C exhibited larger compared with other temperatures, as shown in Fig. 8(a–c), suggesting that the EIS devices after RTA at higher temperature produce low bulk resistances. On the other hand, the diameter of the semicircle was almost the same value as the accumulation, depletion, and inversion regions. Figure 9(a–c) depict that the Nyquist plots of the YbTaO_4_ EIS sensors after RTA at three temperatures were performed under three regions and then tested at pH 7. Here, a lower real impedance value (small semicircle) occurred at higher frequency, whereas a higher real impedance value (large semicircle) appeared at lower frequency. The impedance spectra (Z_im_ vs. Z_re_) of the YbTaO_4_ EIS sensors annealed at the three temperatures and then tested at pH 10 under the three regions were shown in Fig. 10(a–c). The radius of the semicircles for these EIS sensors gradually decreased from ~3–3.6 to ~1.8–2.1 kΩ as the RTA temperature increased. Moreover, the radius of the semicircle in accumulation region was higher than those of depletion and inversion regions. Figures 8 and 10 demonstrate that the radius of the semicircles for the EIS sensors tested in the alkaline (pH 10) solution were higher than those in the acid (pH 4) solution. The size of H_3_O^+^ ions is larger than that of the HO^−^ ions, thus leading to a low diffusion rate. Therefore, the bulk resistance of the EIS devices gradually increased from ~0.4 to ~1 kΩ as the pH value increased.

## Conclusions

The effect of RTA treatment on the structural, sensing and impedance characteristics of YbTaO_4_ sensing films on Si substrates by means of reactive rf cosputtering was explored in the paper. Material analyses indicate that the YbTaO_4_ sensing film after RTA at 800 °C could form a stoichiometric YbTaO_4_ structure and increase the grain size of (−131) plane. The YbTaO_4_ sensing film annealed at 800 °C exhibited a higher pH sensitivity of 71.17 mV/pH, a smaller hysteresis voltage of 1 mV and a lower drift rate of 0.22 mV/h, in comparison with other RTA temperatures. These results are attributed this RTA temperature to the reduction in the defects and the improvement in the material quality of the film and the interface of YbTaO_4_-Si substrate. The semicircle radius of the Nyquist plot for YbTaO_4_ EIS device performed under the accumulation region was larger than those under the depletion and inversion regions. Furthermore, the bulk resistance gradually decreased with increasing the RTA temperature, while it clearly increased with increasing the pH value. The YbTaO_4_-based EIS sensor annealed at 800 °C is suitable for use in future medical and industrial biosensing applications.
